# Comparison between the double‐syringe and the single‐syringe techniques of adenosine administration for terminating supraventricular tachycardia: A pilot, randomized controlled trial

**DOI:** 10.1002/clc.23820

**Published:** 2022-03-27

**Authors:** Praew Kotruchin, Itchaya‐on Chaiyakhan, Phimonphorn Kamonsri, Wittawin Chantapoh, Nattapat Serewiwattana, Nayawadee Kaweenattayanon, Nattacha Narangsiya, Piyangkul Lorcharassriwong, Kittithat Korsakul, Punnapat Thawepornpuriphong, Tanachoke Tirapuritorn, Thapanawong Mitsungnern

**Affiliations:** ^1^ Department of Emergency Medicine, Faculty of Medicine Khon Kaen University Muang Khon Kaen Thailand; ^2^ Emergency Medicine Unit Srisaket Hospital Muang Srisaket Thailand; ^3^ Kukan Hospital Srisaket Thailand; ^4^ Emergency Medicine Unit Queen Sirikit Heart Center of the Northeast Khon Kaen Thailand; ^5^ Emergency Medicine Unit Khon Kaen Hospital Muang Khon Kaen Thailand; ^6^ Yangchumnoi Hospital Srisaket Thailand; ^7^ Kalasin Hospital Kalasin Thailand; ^8^ Jun Hospital Payao Thailand; ^9^ Wisetchaicharn Hospital Angthong Thailand

**Keywords:** adenosine, efficacy and side effects, single‐syringe technique, supraventricular tachycardia

## Abstract

**Background:**

Adenosine has been recommended as a first‐line treatment for stable supraventricular tachycardia (SVT). Standard guidelines recommend 6‐mg of adenosine administered intravenously (IV) with an immediate 20‐ml IV bolus of normal saline solution (NSS; double syringe technique [DST]). However, a newly proposed single‐syringe technique (SST), in which adenosine is diluted with an up to 20 ml IV bolus of NSS, was found to be beneficial.

**Hypothesis:**

We hypothesized that the SST was noninferior to the DST for terminating stable SVT.

**Methods:**

A pilot multicenter, single‐blind, randomized controlled study was conducted at nine hospitals in north and northeast Thailand. Thirty patients who were diagnosed with stable SVT were randomized into two groups of 15, with one receiving adenosine via the DST and the other via the SST. We examined SVT termination, the average successful dose, and the complication rate of each group. Analyses were based on the intention‐to‐treat principle.

**Result:**

The termination rate was 93.3% in the DST and 100% in the SST group (*p* = 1.000), and the success rate of the first 6‐mg dose of adenosine was 73.3% and 80%, respectively (*p* = 1.000). The total administered dose was 8.6 ± 5.1 mg in the DST group and 7.6 ± 4.5 mg in the SST group (*p* = .608). No complications were found in either group.

**Conclusions:**

The SST was non‐inferior to the DST for termination of SVT. However, a further definitive study with a larger sample size is required.

## 1 INTRODUCTION

Supraventricular tachycardia (SVT) is the most common tachyarrhythmia in young adults, children, and infants.^1^ Treatment of SVT is based on the patient's hemodynamic stability. The vagal maneuvers and/or pharmaceutical therapy are recommended for hemodynamically stable patients.^2^ Studies have reported the effectiveness of vagal maneuvers (carotid sinus massage) in SVT termination to be around 19%–54%.^2^ The relatively low success rate of vagal maneuvers makes adenosine crucial in terminating SVT.

The standard guidelines for management of SVT state that adenosine should be the first medication therapy if vagal maneuvers fail to terminate SVT.^2^ A recent systematic review showed that adenosine was not only equally efficacious in treating SVT but was also safer than intravenous calcium channel blockers (CCB).^3^ Furthermore, another study found adenosine to be a safe and effective treatment for SVT in pre‐hospital settings.^4^ It has a success rate of over 90% for terminating SVT.^5^, ^6^

Adenosine prolongs atrioventricular (AV) conduction and results in a transient AV block that is responsible for reentrant tachyarrhythmia termination.^5^, ^6^, ^7^ Dosage via the peripheral intravascular (IV) route is 6 mg, followed by 12 mg if the first dose is ineffective.^2^ To achieve rhythm conversion, IV administration should be performed using a rapid bolus with an immediate normal saline solution (NSS) flush through a large vein (e.g., the antecubital vein) within 1–2 s.^7^, ^8^ This conventional method of adenosine administration is called the double syringe technique (DST).^2^ This method generally requires an IV line and/or a T‐way stopcock, and two syringes.^9^ Furthermore, at least two nursing staff are needed to administer the adenosine and NSS simultaneously to ensure effectiveness.^9^ In real‐world practice, small or rural hospitals may have a shortage of medical staff and equipment, resulting the DST being performed improperly. A more convenient single syringe technique (SST), has thus been proposed, in which a bolus of adenosine is mixed with NSS up to 15–20 ml and administered intravenously.^10^ In a previous nonblind randomized prospective study, this method resulted in a higher success rate than the DST (85.7 vs. 80%), but the difference was not statistically significant.^11^ Another observational study found that the SST resulted in a similar conversion success rate to that of the DST.^10^ However, to the best of our knowledge, no randomized controlled trial (RCT) with a blind protocol has been conducted to confirm this result. We thus conducted this multicenter RCT to evaluate the non‐inferiority of the SST compared with the DST in terminating stable SVT.

## 2 METHODS

### 2.1 Study design, study setting, and population

A pilot, multicenter, single‐blind, RCT was conducted from January to December 2021. We enrolled 32 patients aged between 18 and 80 years at the emergency rooms of nine hospitals in Thailand (Srinagarind Hospital, Queen Sirikit Heart Center of the Northeast, Srisaket Hospital, Khon Kaen Hospital, Yangchumnoi Hospital, Kukan Hospital, Kalasin Hospital, Wisetchaicharn Hospital, and Jun Hospital).

We included patients who presented with regular rhythm, narrow QRS complex tachyarrhythmias (QRS complex <0.12 ms), and heart rate (HR) ≥ 150 beats per minute (bpm) without P wave or with retrograde P wave. A 12‐lead ECG was immediately reviewed by a cardiologist to confirm the diagnosis of SVT before enrollment.

We excluded cardiac arrest patients, patients with a history adenosine allergy, patients who had concurrent acute asthmatic attacks, patients with signs of hemodynamic instability (including chest pain, heart failure, alteration of consciousness, hypotension, or signs of shock), patients with known or probability of pregnancy, and those with unsuccessful IV access at the cubital vein.

For safety reasons, patients were prematurely terminated from the study if they developed any serious adverse effects from adenosine, they had worsening hemodynamic status at any time after enrollment, or the ECG revealed a rhythm other than SVT after adenosine administration such as atrial flutter or atrial tachycardia.

The study was to be terminated prematurely if there was a difference in success rate between the two arms larger than 20% at any time according to the interim analyses.

The Khon Kaen University Ethics Committee in Human Research approved the study. (HE631158) All patients provided written informed consent before enrollment. The study was pre‐registered to ClinicalTrials.gov (NCT05022290).

### 2.2 Definition of SVT termination

Termination of SVT was defined as ECG findings indicating a sinus rhythm with the presence of an upright P wave in lead II (normal P axis, 0 to +90°) and a P wave observed preceding each QRS complex^12^ after the administration of adenosine.

### 2.3 Sample size calculation

To compare the success rate of SVT termination between the two methods, a sample size was calculated for a one‐tailed test with an alpha error probability of .05, power of 0.8, and a noninferiority margin of 20%. In a previous study, the success rates of the DST and SST were 80% and 85.7%, respectively.^11^ To prove the hypothesis that SST is noninferior to DST, 350 patients were needed in each arm. However, due to the emergence of the COVID‐19 pandemic, there was significant concern regarding patient safety, and the trial was put on hold for 6 months by regional lockdowns, this population size would not be achievable within planned study period. As a result, we decided to conduct a pilot study to obtain preliminary results before proceeding further with the confirmatory RCT. To achieve normally distributed data, a sample size of 30 participants—15 in each arm—were enrolled to compare the efficacy of the SST with that of the DST.

### 2.4 Randomization and study protocol

After filtering patients based on the inclusion and exclusion criteria, those who remained were given details of the study and enrolled after they provided written informed consent. Every patient was provided treatment according to the standard of care in the ER including measurement of vital signs, ECG monitoring, IV line placement at the cubital vein, and blood testing. Subsequently, the attending physicians contacted a cardiologist to confirm the diagnosis of SVT and then the randomization center was contacted by phone to allocate each patient into either the DST or SST group. The center used sequentially numbered, opaque, sealed envelopes (SNOSE) to ensure allocation concealment, and then told the attending physician to provide therapy according to the patient's allocated group. The randomization numbers were generated using computerized block randomization (block of four; www.sealedenvelope.com) with an allocation ratio of 1:1. The author conducting analysis was blinded, but the attending physician and patient were not.

### 2.5 Adenosine administration

#### 2.5.1 Control (DST) group

**Figure 1:**
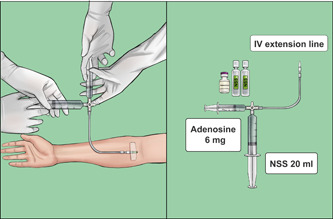
The double‐syringe technique (DST) for adenosine administration. Adenosine (6 mg per 2 ml) in a 5‐ml syringe with a subsequent 20 ml bolus of normal saline solution (NSS) in another 20‐ml syringe connected by a T‐way stopcock were administered via the cubital vein. If this failed to terminate supraventricular tachycardia (SVT), a second dose of adenosine (12 mg per 4 ml) in a 5 ml syringe was given using the same technique.

Patients randomized into the DST group received the first dose of adenosine (6 mg per 2 ml) in a 5‐ml syringe with a subsequent 20 ml bolus of NSS in another 20‐ml syringe connected by a T‐way stopcock via the cubital vein (Figure 1). If SVT termination failed, a second dose of adenosine (12 mg per 4 ml) in a 5 ml syringe was administered using the same technique. If the second dose was unsuccessful, we considered the treatment to have failed. In such cases, the patient received further standard treatment based on their hemodynamic status including other medication (e.g., nondihydropyridine CCB) or electrical cardioversion.

#### 2.5.2 Intervention (SST) group

**Figure 2:**
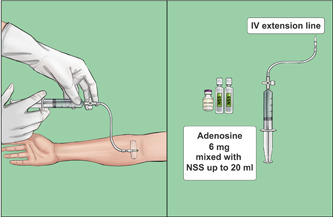
The single‐syringe technique (SST) for adenosine administration. A rapid bolus of adenosine (6 mg per 2 ml) mixed with up to 20 ml of normal saline solution (NSS) in a 20‐ml syringe was intravenously within 1–2 s via the cubital vein. If this failed to terminate supraventricular tachycardia (SVT), a second dose of adenosine (12 mg per 4 ml) mixed with up to 20 ml of NSS was administered using the same technique

For the SST group, we used rapid bolus injection to administer the first dose of adenosine (6 mg per 2 ml) mixed with up to 20 ml of NSS in a 20‐ml syringe within 1–2 s via the cubital vein (Figure 2). If SVT termination failed, we administered another dose of adenosine (12 mg per 4 ml) mixed with up to 20 ml of NSS using the same technique. If the second dose was unsuccessful, we considered the treatment to have failed. Patients in whom the treatment failed were given subsequent treatment including other medication (e.g., nondihydropyridine CCB) or electrical cardioversion.

### 2.6 Outcome measures and statistical analysis

The primary outcome was the success rate of SVT termination, determined by ECG rhythm strip (lead II) and/or 12‐lead ECG interpreted by an experienced cardiologist, which was defined within 3 min after administering each dose of adenosine or before the next treatment was ordered. The secondary outcomes were the average total dose of adenosine required to terminate SVT and the rate of adverse events compared between groups.

Descriptive data were shown as number, percentage, mean, and standard deviation. Analytical data were demonstrated using an inferiority test with a 95% confidence interval (CI) applying the principle of intention to treat analysis. Statistical significance was defined as *p* < .05. SPSS version 26 and STATA version 10.1 were used for these analyses.

## 3 RESULTS

**Figure 3:**
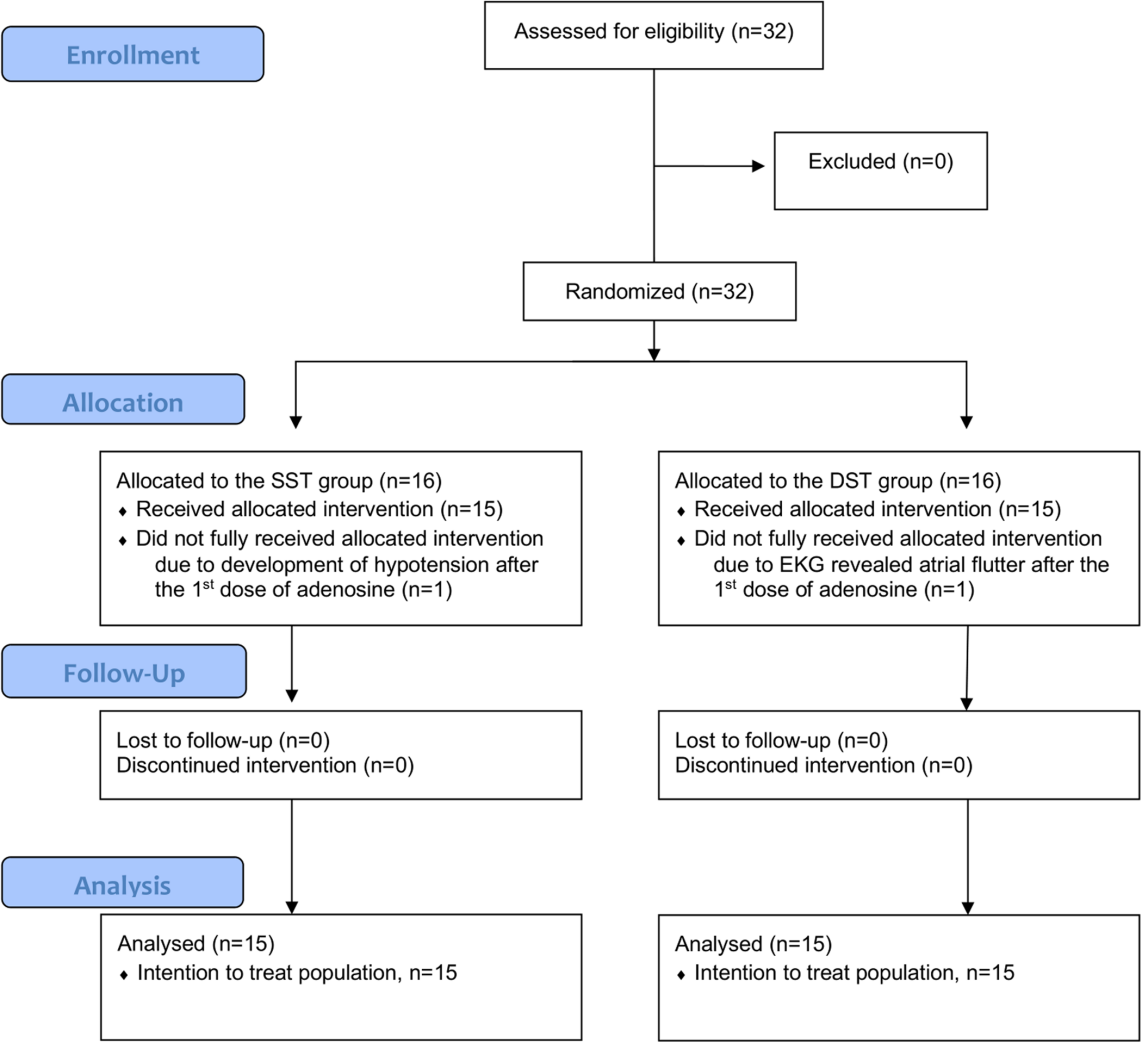
The CONSORT flow diagram. Thirty‐two patients were enrolled. Sixteen patients were randomized into the double syringe technique (DST) group, one of whom was later withdrawn because their electrocardiogram (ECG) revealed atrial flutter after the first dose of adenosine. Sixteen patients were randomized to the single‐syringe technique (SST) group, one of whom was withdrawn due to a hemodynamic deterioration (developed hypotension) after adenosine treatment. The remaining 15 patients in each group were included in the final analysis.

There was a total of 32 patients enrolled in the study, 16 in each the DST and SST group. However, two patients were withdrawn, one in the DST group because their ECG revealed atrial flutter after the first dose of adenosine and another in the SST group due to hemodynamic deterioration (developed hypotension) after the first dose of adenosine. This left 15 patients in each group to be included in the final analysis (Figure 3).

### 3.1 Demographic data

The average age of the total study population was 54 years (years) and did not differ between the two groups (DST: 54.5 ± 14.0 years; SST: 54.8 ± 20.9 years). Women predominated in both groups (DST: 73.3% and SST: 66.7%). However, the DST group had a higher average BMI (24.0 ± 4.2 kg/m^2^ vs. 22.0 ± 3.3 kg/m^2^). The most common chief complaint in both groups was palpitation (73.3% and 93.3% in the DST and SST group, respectively). The average initial HR of the DST group was slightly higher at 169 bpm versus 164 bpm, as was posttreatment HR (99 bpm vs. 92 bpm). Serum creatinine was also higher in the DST group at 1.05 ± 0.62 mg/dl versus 0.90 ± 0.23 mg/dl (Table 1).

**Table 1:** Baseline characteristics

	Total (*N* = 30)	Single‐syringe group (*n* = 15)	Double‐syringe group (*n* = 15)
Age, years	54.6 ± 17.4	54.8 ± 20.9	54.5 ± 14.0
Men, *n* (%)	9 (30)	5 (33.3)	4 (26.7)
BMI, kg/m^2^	23.0 ± 3.8	22.0 ± 3.3	24.0 ± 4.2
Chief complaint			
Palpitation, *n* (%)	25 (83.3)	14 (93)	11 (73.3)
Fatigue, *n* (%)	2 (6.7)	1 (6.7)	1 (6.7)
Near syncope, *n* (%)	1 (3.3)	0 (0)	1 (0)
Physical stress, *n* (%)	2 (6.7)	0 (0)	2 (0)
Onset of symptoms to ER, hours	5.6 ± 8.5	4.7 ± 9.0	5.4 ± 8.4
Initial vital signs			
BT, Celsius	36.7 ± 1.1	36.4 ± 0.5	37.0 ± 1.4
SBP, mmHg	125 ± 22	122 ± 17	130 ± 25
DBP, mmHg	79 ± 15	79 ± 15	81 ± 18
HR, bpm	164 ± 27	164 ± 27	169 ± 18
Posttreatment vital signs			
BT, Celsius	36.7 ± 0.78	36.5 ± 0.4	36.9 ± 1.0
SBP, mmHg	119 ± 18	123 ± 18	116 ± 17
DBP, mmHg	73 ± 10	76 ± 7	71 ± 12
HR, bpm	95 ± 15	92 ± 11	99 ± 18
Creatinine, mg/dl	0.98 ± 0.47	0.90 ± 0.23	1.05 ± 0.62
Potassium, mEq/L	3.84 ± 0.52	3.85 ± 0.63	3.82 ± 0.44

Abbreviations: BMI, body mass index; BT, body temperature; DBP, diastolic blood pressure; HR, heart rate; SBP, systolic blood pressure.

### 3.2 Primary and secondary outcomes

The termination rate was 93.3% in the DST and 100% in the SST group (*p* = 1.000), and the success rate of the first 6‐mg dose of adenosine was 73.3% and 80%, respectively (*p* = 1.000). The total administered dose was 8.6 ± 5.1 mg in the DST group and 7.6 ± 4.5 mg in the SST group (*p* = .608). In the SST group, there was a certain amount of adenosine that remained in the extension tube (1.8‐ml dead cavity). Therefore, 0.54 mg of the first 6‐mg dose, and 1.08 mg of the 12‐mg second dose was deducted. No adverse events were observed in either group (Table 2).

**Table 2:** Comparison of the outcomes between the single‐syringe technique and the double‐syringe technique of adenosine administration

Outcomes	Single‐syringe group (*n* = 15)	Double‐syringe group (*n* = 15)	*p*
SVT termination, *n* (%)	15 (100)	14 (93.3)	1.000
SVT termination at first dose (6 mg) of adenosine, *n* (%)	12 (80)	11 (73.3)	1.000
Total dose of adenosine, mg	7.6 ± 4.5	8.6 ± 5.1	.608
Discharge, *n* (%)	12 (80)	10 (66.7)	.682
Major adverse event, *n* (%)	0 (0)	0 (0)	1.000
Home medication			
CCB, *n* (%)	7 (46.7)	7 (46.7)	NA
Beta‐blockers, *n* (%)	1 (6.7)	1 (6.7)	
Other drugs, *n* (%)	1 (6.7)	0 (0)	
Unknown, *n* (%)	6 (40)	7 (46.7)	

Abbreviations: CCB, calcium channel blockers; SVT, supraventricular tachycardia.

Ten of 15 patients in the DST group and 12 of 15 in the SST group were discharged. None of the cases in which admission was required were due to SVT. These patients had other concomitant diseases or conditions. The types of home medications prescribed in each group were similar, the most common being non‐dihydropyridine CCB (46.7% in both groups; Table 2).

## 4 DISCUSSION

This pilot randomized controlled trial showed that the novel adenosine administration method—namely, the single‐syringe technique or SST—was noninferior to the conventional method, known as the double‐syringe technique (DST) in terminating SVT. In addition, neither method caused any adverse events in this study.

In our population of SVT sufferers, the number of women was twice that of men. This is consistent with the epidemiology of SVT (especially AVNRT) in the general population, which was reported as being more common in women.^1^ Patient age in our study was similar to that in studies by McDowell et al.^10^ and Goyal et al.,^13^ in which the mean age was 54–58 years. However, a study by Choi et al.^11^ reported a lower average age of SVT patients in Korea (mean age of 48 years). The most common chief complaint of patients in our study was palpitation, which is consistent with previous studies.^14^ Average HR was 164 bpm, which is also within the usual range (120–220 bpm) in SVT.^14^ Mean serum potassium in this study was within the normal limits. Due to the pathophysiology of SVT (reentrant mechanism),^14^ patients' serum electrolytes are generally normal, and unlike other arrhythmias (early after depolarization of the triggered arrhythmias mechanism), electrolyte abnormalities have relatively little effect on SVT initiation.^15^

In terms of treatment outcomes, we found that the rate of SVT termination was non‐significantly higher in the SST group than in the DST group (100% vs. 93.3%, *p* = 1.0). This result was consistent with a nonblind, randomized prospective study conducted by Choi et al.,^11^ which found a higher successful termination rate using SST method (SST 85.7% vs. DST 80%, *p* = .390). The findings were also in line with an observational study by McDowell et al.,^10^ which demonstrated that SST resulted in a higher success rate after the first dose (6 mg) than DST (73.1% vs. 40%, *p* = .017). Furthermore, the total average dosage of adenosine required for SVT termination in the SST group was slightly lower than that of the DST group (7.6 vs. 8.6 mg, *p* = .928). These results were consistent with those of McDowell et al.'s study, which found the total dosage used to be slightly lower in the SST group (SST 10.3 mg vs. DST 11 mg).^10^ However, they contrasted with those of Choi et al.,^11^ who found that the total average dose of adenosine in the SST group was slightly higher than in the DST group (SST 11.0 mg vs. DST 10.3 mg, *p* = .070).

We did not observe any adverse events in either group, which was consistent with both studies mentioned above.^10^, ^11^ However, adenosine may theoretically cause side effects such as transient AV block, flushing, chest pain, hypotension, dyspnea, atrial fibrillation (AF), premature ventricular tachycardias (PVCs) or ventricular tachycardia (VT), bronchospasm (rare), or coronary steal.^16^, ^17^, ^18^, ^19^ We excluded patients with concomitant acute asthmatic attack, and patients with history of adenosine allergy to minimize the risk of any possible adverse events mentioned above. We found that neither the DST nor the SST caused new‐onset AF.

One strength of this study was that it was an RCT, which minimized the possibility of selection bias. Second, the 12‐lead ECG was interpreted by experienced cardiologists to exclude other narrow regular tachyarrhythmias (e.g., atrial tachycardia, sinus tachycardia, and atrial flutter). Furthermore, the primary outcome was clinically important. Instead of using RR‐interval widening as an outcome, we defined “success” as a complete termination of SVT, or in other words, conversion to a normal sinus rhythm. However, there were some potential limitations. First, due to the small number of patients that we enrolled in this pilot study, the study may have lacked the power to make a firm conclusion regarding the treatment effect. Therefore, a further definitive study with larger sample size is needed to prove the non‐inferiority or superiority of the SST to the DST for termination of SVT. Second, we did not measure the intravenous flow rate of adenosine delivery which might also have an impact on termination of SVT. Furthermore, the use of an extension tube to administer adenosine in the SST group caused a 1.8‐ml dead cavity with the remaining dose of adenosine outside body system. Although a total dose for SVT termination was not different between the DST and the SST group even after we deducted the remaining dose in the dead cavity by calculation. In the future definitive study, we suggest either to inject more amount of adenosine, or to administer the medication into the antecubital vein directly via a T‐way stopcock without an extension tube in the SST arm. Finally, the study was conducted in Thai patients, and care should be taken when extrapolating the results to other populations.

## 5 CONCLUSIONS

Adenosine diluted with up to 20 ml of NSS administered intravenously (SST) was non‐inferior to the conventional technique (DST) for terminating SVT. The SST was also practical and safe. However, a further definitive study with larger sample size is required.

## CONFLICTS OF INTEREST

The authors declare no conflicts of interest.

## AUTHOR CONTRIBUTIONS

Praew Kotruchin conceived and designed the analysis, and interpreted the data. Itchaya‐on Chaiyakhan drafted the manuscript and was responsible for acquisition of funding. Thapanawong Mitsungnern performed the critical revision of the manuscript. Phimonphorn Kamonsri, Wittawin Chantapoh, Nattapat Serewiwattana, Nayawadee Kaweenattayanon, Nattacha Narangsiya, Piyangkul Lorcharassriwong, Kittithat Korsakul, Punnapat Thawepornpuriphong, and Tanachoke Tirapuritorn contributed in the acquisition of the data.

## Data Availability

The data that support the findings of this study are available on request from the corresponding author (TM).
